# Predicted functional alterations in colonic microbiota metabolism underlie ethanol consumption and preference behavior in mice

**DOI:** 10.1111/acer.70165

**Published:** 2025-10-30

**Authors:** Mírian Velten Mendes, Thiago Cavalcante Lima, Mariana Siqueira Amormino, Jamil Silvano de Oliveira, Fernanda Lima Alvarenga Barroso, Gaëlle Boudry, Renato Elias Moreira‐Júnior, Ana Lúcia Brunialti‐Godard

**Affiliations:** ^1^ Laboratório de Genética Animal e Humana, Departamento de Genética, Ecologia e Evolução, Instituto de Ciências Biológicas Universidade Federal de Minas Gerais Belo Horizonte Brazil; ^2^ Departamento de Bioquímica e Imunologia, Instituto de Ciências Biológicas Universidade Federal de Minas Gerais Belo Horizonte Brazil; ^3^ Institut NuMeCan, INRAE, INSERM University Rennes Rennes France

**Keywords:** alcohol use disorder, dysbiosis, ethanol preference, gut microbiota, metabolites

## Abstract

**Background:**

Alcohol use disorder (AUD) is a complex condition affecting several body systems. Gut microbiota alterations, intestinal‐barrier disruption, and the consequent translocation of metabolites foster chronic inflammation, lower short‐chain fatty acid (SCFA) output, and depleted beneficial bacteria may contribute to transcriptional, epigenetic, and metabolic changes that influence ethanol preference.

**Methods:**

Two experimental phases were used. T1 (8 weeks): mice received either the American Institute of Nutrition standard diet (AING) or a high‐sugar‐butter (HSB) diet. T2 (4 weeks): HSB animals switched to AING (SWITCH), while AING mice maintained the same diet. Each diet arm was split into ethanol (EtOH; free access to 10% ethanol) or H_2_O, generating four groups (AING + H_2_O, AING + EtOH, SWITCH + H_2_O, and SWITCH + EtOH). Sample processing involved colonic‐content collection, 16S rRNA sequencing, microbiome profiling, functional inference, metabolic‐network analysis, and SCFA/amino acid quantification.

**Results:**

SWITCH + EtOH mice displayed high ethanol consumption and preference, whereas AING + EtOH mice showed ethanol aversion. Their colonic microbiota differed markedly; amino acid metabolism fell, secondary bile acid synthesis rose, and SCFA production dropped in SWITCH + EtOH animals. Direct measurements confirmed significant reductions in butyrate, acetate, propionate, and selected amino acids. Network analysis revealed enrichment of bacterial metabolism, oxidative stress, and dopamine pathway genes.

**Conclusions:**

Diet‐induced dysbiosis, reflected in shifts in microbiota‐derived metabolites, was associated with excessive alcohol intake; the metabolites identified can represent potential therapeutic targets for AUD.

## Graphical abstract

Using a free‐choice ethanol model, we show that mice switched from a high‐sugar/butter diet to AIN93G standard diet consume and prefer more alcohol than mice maintained on AIN93G. 16S profiling and inferred metabolome revealed reduced amino acid metabolism, increased secondary bile acid pathways, and lower microbiota‐derived SCFAs and selected amino acids. Network analyses highlighted bacterial metabolism, oxidative stress, and dopamine‐related genes. These metabolite shifts link diet‐driven microbiota changes to excessive drinking and nominate microbiota‐derived metabolites as therapeutic targets for AUD.

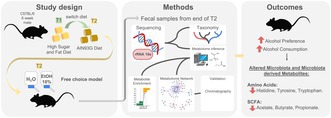

## INTRODUCTION

Alcohol use disorder (AUD) is defined as a pathological condition characterized by problematic alcohol consumption that leads to clinically significant consequences (American Psychiatric Association, DSM‐5 Task Force, 2013). These may include persistent and uncontrollable cravings for alcohol despite adverse outcomes, abandonment of social activities, the development of tolerance, and the manifestation of withdrawal syndrome. Moreover, alcohol consumption is widely recognized as a contributing factor to numerous health issues, such as cardiovascular disease, hypertension, type 2 diabetes mellitus, alcoholic liver disease, and increased susceptibility to bacterial and viral infections (Biddinger et al., 2022; Szabo & Saha, 2015). Despite the well‐established risks, alcohol remains legal for adults in most countries. According to the World Health Organization (WHO), “No level of alcohol consumption is safe for our health,” yet its prevalence in society remains high.

At the molecular level, alcohol addiction results from complex and intersecting pathways affecting mood, motivation, and stress circuits in the basal ganglia, extended amygdala, habenula, prefrontal cortex (PFC), insula, and allocortex (Koob & Volkow, 2016). Furthermore, addiction involves the interplay of several neurochemical circuits that progress through three main stages: (1) a positive reinforcement stage, mediated by increased activity of dopamine, opioid peptides, histamine, γ‐aminobutyric acid (GABA), and acetylcholine; (2) a negative reinforcement stage, arising from a decrease in reward‐system sensitivity and an increase in corticotropin‐releasing factor, norepinephrine, and dynorphin; and (3) an anticipation (craving) stage, driven by glutamate signaling and disrupted GABAergic activity in the PFC (Koob & Volkow, 2016).

An emerging area of AUD research focuses on its relationship with the gut microbiota essential to human health homeostasis, regulating host immune responses and metabolism through multiple mechanisms (Visconti et al., 2019). The gut microbiota maintains bidirectional communication with the brain via the gut–brain axis, enabling mutual influence and affecting both host behavior and the development of brain disorders (Morais et al., 2021). Consequently, any disturbance in microbial community balance or altered brain chemistry can produce various adverse effects (Chen et al., 2022). Under these circumstances, the relationship between dysbiosis and alcohol consumption proves to be highly complex. It has been demonstrated that the microbiome from individuals with AUD can trigger alcohol cravings in rat models and that gut microbial metabolites may permeate the intestinal barrier due to alcohol‐induced leaky gut, leading to generalized neuroinflammation and exacerbating psychiatric symptoms in AUD (Leclercq et al., 2012; Wang et al., 2024). Metabolites such as neurotransmitters (e.g., GABA, dopamine, and serotonin) are significantly altered in heavy alcohol consumption due to dysbiosis (Chen & Xu, 2021). In humans with AUD, several commensal gut microbes, including short‐chain fatty acid (SCFA)‐producing bacteria, are altered, causing shifts in metabolites and further increasing gut permeability (Dubinkina et al., 2017; Wang et al., 2024). Patients exhibiting higher gut permeability show more pronounced psychological withdrawal symptoms, indicating that gut microorganisms, through their impacts on gut homeostasis, can significantly influence behavioral outcomes (Leclercq et al., 2014). Furthermore, alcohol consumption alters serum amino acid (AA) levels and reshapes gut microbial populations involved in their metabolism, degradation, and synthesis (Kawase et al., 2017; Neis et al., 2015). This aspect is essential because AAs can serve as precursors for SCFAs and are indispensable for synthesizing neurotransmitters, such as histamine, gamma‐aminobutyric acid (GABA), and dopamine (Kawase et al., 2017; Neis et al., 2015). SCFAs maintain gut barrier integrity, modulating immune responses and inflammatory processes, while the availability of AAs, partly regulated by the microbiota, affects metabolic and synaptic pathways (Kawase et al., 2017; Silva et al., 2020). This underscores the close relationship between microbiota, metabolism, and behavior in AUD.

Animal models serve as valuable tools to investigate how microbiota alterations influence alcohol consumption, preference, and host metabolism. In this context, our research group developed an animal model characterized by high ethanol consumption and preference (Martins de Carvalho et al., 2018). In this model, mice are initially fed a high‐sugar butter diet (HSB), which is replaced with a standard (AING93‐G) diet, while a 10% ethanol solution is freely available alongside water (Martins de Carvalho et al., 2018). The transition from the HSB to the standard diet leads to high ethanol intake and a preference for ethanol over water, likely due to HSB consumption‐induced desensitization of the dopaminergic reward system and the accompanying dysbiosis, leading to alcohol consumption as a compensatory mechanism for an impaired reward system (Martins de Carvalho et al., 2018). In contrast, mice not exposed to the HSB diet do not develop ethanol preference and exhibit aversion (Martins de Carvalho et al., 2018; Moreira Júnior et al., 2019, 2023). We hypothesize that a high preference for ethanol is associated with broad alterations in colonic microbiota and its metabolites, changes that may modulate alcohol consumption behavior or emerge as its consequence. Supporting this hypothesis, Moreira Júnior et al. (2019) demonstrated that mice transitioning from the HSB to the AIN93‐G diet and developing ethanol preference exhibited a significantly different colonic microbiota profile compared with those showing ethanol aversion (Moreira Júnior et al., 2019). These differences included increased microbial diversity, higher Bacillota and Actinomycetota groups, and significant changes in the abundance of bacterial families involved in bioactive metabolite production (Moreira Júnior et al., 2019). Given these initial findings, this study aimed to investigate the functional alterations in microbiota‐derived metabolite production in mice with high ethanol preference and to elucidate the metabolic mechanisms that could modulate excessive ethanol consumption and preference compared with aversion.

## METHODOLOGY

### Animals and experimental design

**FIGURE 1:**
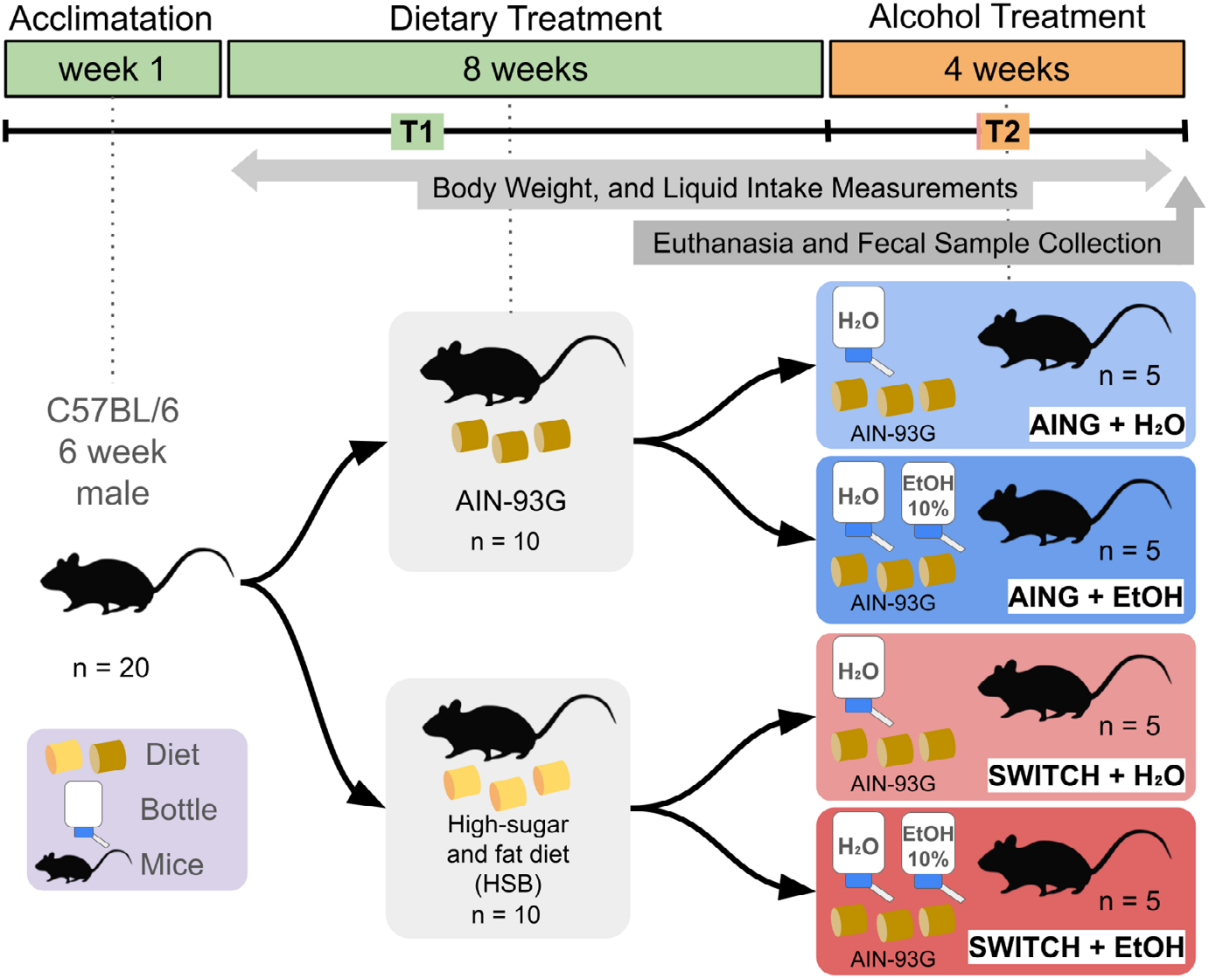
Experimental design of the free‐choice alcohol model in mice. Following a one‐week acclimation period, 20 C57BL/6 mice were assigned to two dietary regimens for 8 weeks (T1): The AING diet or a high‐sugar and butter (HSB) diet. At the onset of T2 (4 weeks), each dietary group was subdivided into two, yielding four experimental groups (AING + H_2_O, AING + EtOH, SWITCH + H_2_O, and SWITCH + EtOH; *n* = 5 per group). Mice in the AING + H_2_O and AING + EtOH subgroups continued the AING diet, whereas those in the SWITCH subgroups had their HSB diet replaced with AING. The EtOH subgroups were given free access to both water and 10% ethanol, whereas the H_2_O subgroups were given only water. All mice were euthanized, and samples were collected for further analysis at the end of T2.

For this study, 20 C57BL/6 mice were allocated into four groups—AING + H_2_O (*n* = 5), AING + EtOH (*n* = 5), SWITCH + H_2_O (*n* = 5), and SWITCH + EtOH (*n* = 5), as described by Moreira Júnior et al. (2019) and illustrated in Figure 1. The experiment was divided into two phases: T1 (8 weeks) and T2 (4 weeks). During T1, animals in the AING groups (AING + H_2_O and AING + EtOH) received the American Institute of Nutrition standard diet (AING), while those in the SWITCH groups (SWITCH + H_2_O and SWITCH + EtOH) were fed a high‐sugar and butter (HSB) diet. At the start of T2, the AING groups continued the AING diet, and the SWITCH groups had their diet changed from HSB to AING. The EtOH groups (AING + EtOH and SWITCH + EtOH) were also subjected to a free‐choice protocol with a bottle of 10% ethanol solution and a bottle of water in their home cages, while the H_2_O groups (AING + H_2_O and SWITCH + H_2_O) received only water. Mice were 6 weeks old at the beginning of the experiment and were housed individually under a 12‐hour light/dark cycle. After the 4‐week T2 period, colon content samples were collected from each group. The assessment of body weight, adiposity index, ethanol consumption, and preference was carried out following the study by Martins de Carvalho et al. (2018). All experimental procedures were approved by the Ethics Committee on the Use of Animals of the Federal University of Minas Gerais (CEUA‐UFMG) under protocol numbers 119/2012 and 073/2021. Every effort was made to ensure the well‐being of the animals throughout the experiment.

### Ethanol intake and preference

Water and 10% (v/v) ethanol consumption were measured daily by weighing the drinking bottles before and after a 24‐h period. Intake was expressed as g/kg/day. The ethanol solution was replaced after each measurement. Ethanol preference was considered significant when ethanol consumption exceeded 50.1% of the total fluid intake (*p* < 0.05).

### Sample processing, 16S rRNA sequencing, microbiome profiling, functional inference, and metabolic‐network analysis

Total bacterial DNA was isolated from colonic contents with a silica‐column kit, and the V3–V4 region of the 16S rRNA gene was PCR‐amplified. After bead purification, the amplicons were quantified, equimolarly pooled, and sequenced on an Illumina MiSeq platform. Raw sequences were then quality‐filtered, adapters removed, low‐quality bases trimmed, and reads <100‐bp discarded. The remaining reads were clustered into operational taxonomic units, generating relative‐abundance tables used to calculate α‐diversity (Shannon, Pielou) and β‐diversity (UniFrac distance); α‐diversity was compared by two‐way ANOVA and β‐diversity by PERMANOVA. Taxonomic differences between groups were detected by discriminant analysis. Functional inference mapped sequences to KEGG orthologues, quantifying metabolic pathways enriched or suppressed in animals with high versus low ethanol preference. These results supported the construction of metabolic networks linking orthologues to pathways, and centrality metrics were computed to identify key nodes. A significance threshold of *p* < 0.05 was adopted. All software, versions, and detailed parameters are provided in the Supporting Information.

### Dosage of SCFA and amino acids

SCFA and amino acids from feces were quantified by high‐performance liquid chromatography (HPLC). Briefly, fecal samples (30 mg) were homogenized in 180 μL of ultrapure water adjusted to pH 2.5 using an electric homogenizer for 30 s. Samples were then incubated at room temperature for 10 min, centrifuged at 13,200 rpm for 30 min, and filtered through a hydrophilic nylon syringe filter with a 0.22 μm pore size and 13 mm diameter (Vials, Brazil). Chromatographic analysis was performed on a Nexera UHPLC system (Shimadzu, USA) equipped with a SUPELCOGEL C6‐610H column (Sigma‐Aldrich, USA), injecting 40 μL per run. The mobile phase comprised 0.01 N sulfuric acid at a 0.6 mL/min flow rate, with the column maintained at 45°C. The remaining volumes of the samples previously prepared for SCFA analysis were used for AA analysis. To each sample, 300 μL of acetonitrile were added for protein precipitation. Samples were vigorously vortexed, left to rest for 6 hours at room temperature, and then centrifuged at 16,500 *g* for 10 min in a microcentrifuge (Eppendorf, Centrifuge 5415 C, Germany). The resulting supernatants were collected and evaporated to dryness using a vacuum concentrator (UNIVAPO 100 H, UNIEQUIP, Munich, Germany). Each sample was resuspended in 50 μL of an aqueous solution containing 5 mM sodium acetate (final concentration) and acetonitrile (92:8, v/v), adjusted to pH 7.18. This same solution was used as the mobile phase for chromatographic separation on a Wakosil PTH column (4.6 × 250 mm, Japan), coupled to a UFLC Prominence system (Shimadzu, Japan). A 10 μL aliquot of each sample was injected using an isocratic flow rate of 30 mL/h, with UV detection at 267 and 209 nm and a column temperature set at 39.7°C for the resolution of AA compounds. Acetate, propionate, butyrate, histidine, phenylalanine, tryptophan, and tyrosine were identified by comparison with respective analytical standards.

### Statistical analysis

Statistical analyses were performed using GraphPad Prism software v10.3.1, except for the bioinformatic inferences, which relied on the statistical tests provided by the respective software packages and tools, as previously described. Data distribution was evaluated for normality using the Shapiro–Wilk test. Parametric comparisons employed the two‐tailed Student's *t*‐test, whereas nonparametric data were analyzed using the Mann–Whitney test. If applicable, a two‐way ANOVA was used for multiple‐factor comparisons. A one‐sample t‐test was conducted against the hypothetical value of 50.1% to define alcohol preference. Microbial community structures were visualized through principal coordinates analysis (PCoA) based on unweighted UniFrac distances, with pairwise Adonis and Bonferroni correction applied for between‐group comparisons. Spearman correlations were performed on variables or taxa of interest. Statistical significance was set at *p* < 0.05, and results are reported as mean ± standard error of the mean (SEM). Asterisks denote significance: *p < 0.05, **p < 0.001, ***p < 0.001, ****p < 0.0001.

## RESULTS

### HSB diet intake increases body weight and adiposity index at T1, and its withdrawal leads to high ethanol consumption and preference at T2

**FIGURE 2:**
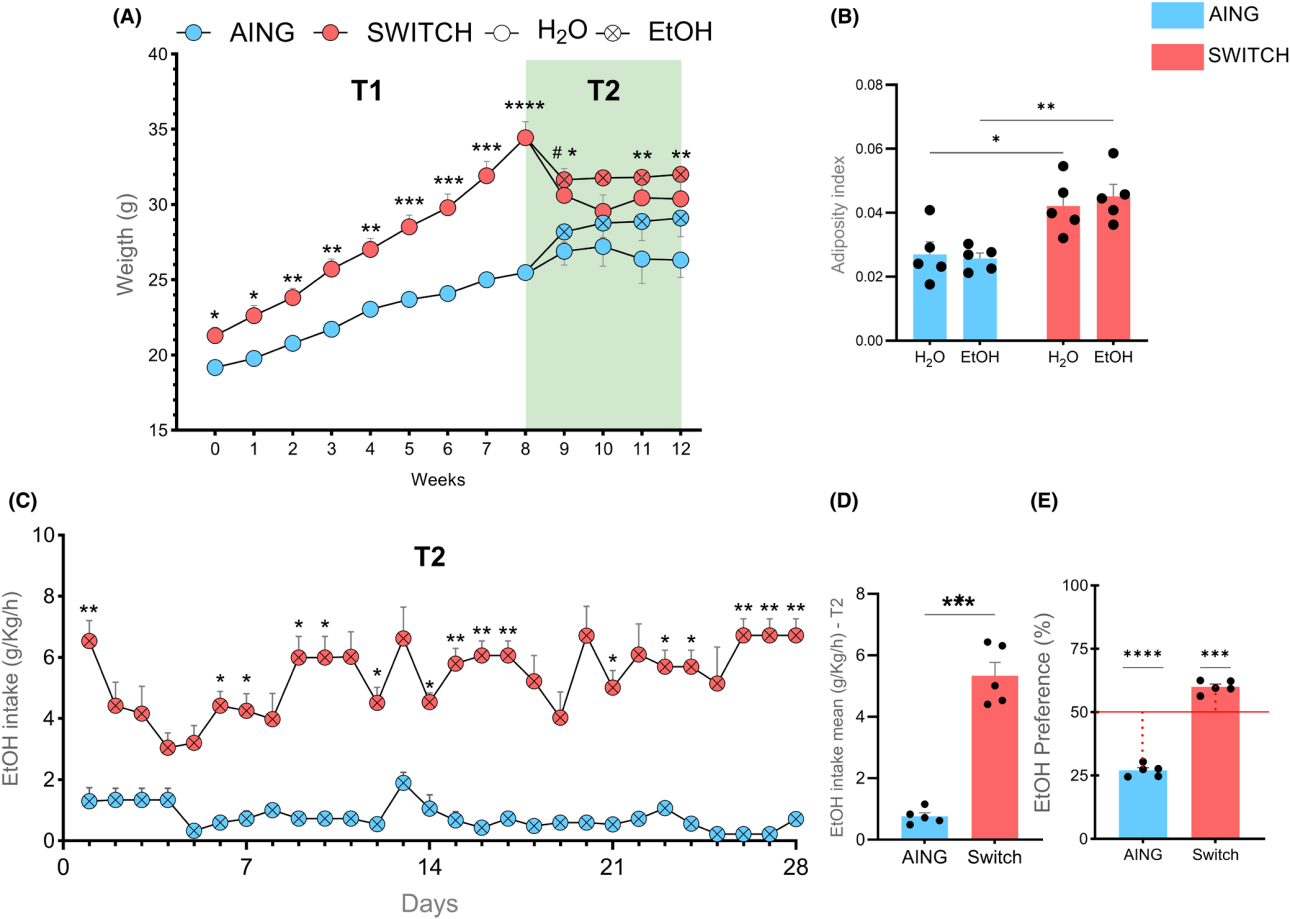
Body weight, adiposity index, ethanol consumption, and ethanol preference. (A) Body weight (g) over T1 and T2. (B) Mean adiposity index at the end of T2. (C) Daily ethanol consumption (g/kg/h) during T2. (D) Mean ethanol consumption (g/kg/h) in T2. (E) Ethanol preference (%). A two‐way repeated measures ANOVA was used to analyze body weight and ethanol intake over time in (A) and (C). A two‐way ANOVA was applied to analyze the adiposity index at the end of T2 in (B). In (D), the Student's *t*‐test was used to compare groups. In (E), a one‐sample *t*‐test was performed against the hypothetical value of 50.1%. Results are expressed as mean ± SEM. Asterisks indicate statistical significance (*p < 0.05, **p < 0.001, ***p < 0.001. ****p < 0.0001, #*p* = 0.0123). In (A), * in T1 indicates differences between AING and SWITCH; in T2, * indicates AING + H_2_O vs. SWITCH + H_2_O and # indicates AING + EtOH vs. SWITCH + EtOH.

During T1, mice fed the HSB diet exhibited a significant increase in body weight (*p* < 0.05) compared with those maintained on the standard AING diet (Figure 2A). In T2, the HSB diet was replaced with the AING diet, establishing the SWITCH groups. Due to the lower caloric density of the new diet (i.e., fewer calories per gram), these mice experienced a reduction in body weight (Figure 2A), with significant differences observed between AING + H_2_O and SWITCH + H_2_O at Weeks 9, 10, and 12 (indicated by *) and between AING + EtOH and SWITCH + EtOH at Week 9 (indicated by #). However, the adiposity index was higher in the SWITCH diet groups (H_2_O and EtOH) compared with their respective controls (H_2_O: *p* = 0.0383, EtOH: *p* = 0.0060) (Figure 2B). Transitioning from the HSB to the AING diet resulted in a significant increase in ethanol intake over several days (*p* < 0.05) and the mean intake across T2 (*p* = 0.000269) in the SWITCH + EtOH group compared with the AING + EtOH group (Figure 2C,D). Furthermore, mice in the SWITCH + EtOH group exhibited a greater preference for ethanol over water (*p* = 0.0009), showing a significant difference from the hypothetical value of 50.1%. In contrast, AING + EtOH mice remained below this threshold, indicating aversion (*p* < 0.0001) (Figure 2E). These findings suggest that mice shifted their preference from the HSB diet to ethanol, indicating the development of an addictive‐like behavior.

### High ethanol consumption and preference behavior lead to a distinct colonic microbiota profile in the SWITCH + EtOH group in comparison to its counterpart AING + EtOH, which showed ethanol consumption aversion

**FIGURE 3:**
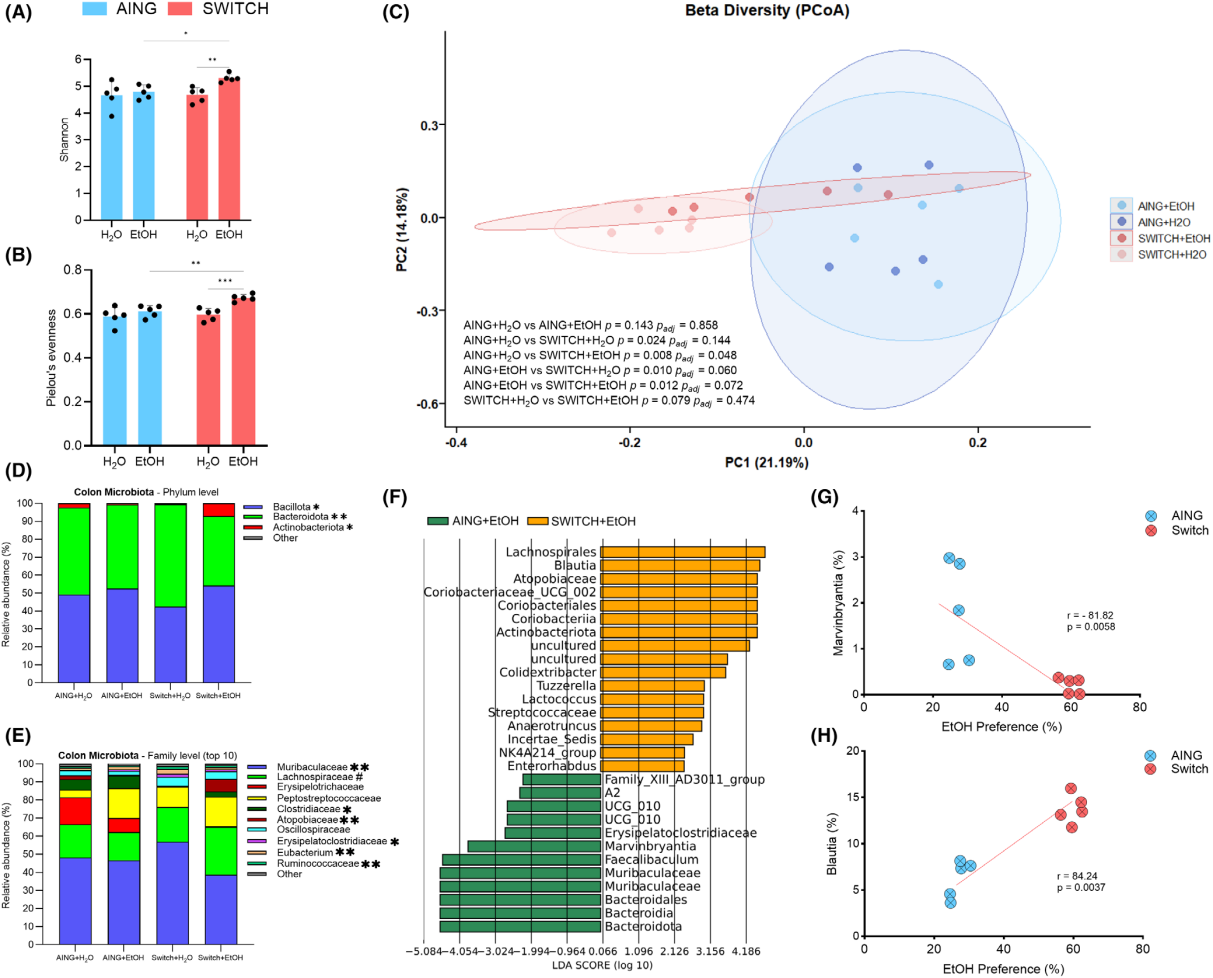
Colonic microbiota analysis at the T2 endpoint. (A) Shannon index and (B) Pielou's evenness (alpha diversity). (C) PCoA of beta diversity. (D) and (E) Taxonomic profiling at phylum and family levels. (F) LDA analysis of ethanol‐exposed groups. (G) and (H) Correlation between ethanol preference and Marvinbryantia or Blautia. A two‐way ANOVA was used to assess alpha diversity in (A) and (B). In (C), the PCoA plot was generated using a distance matrix based on unweighted UniFrac distances, and pairwise Adonis was compared with Bonferroni correction. Taxonomic composition is displayed in (D) and (E). In (F), the LEfSe tool was applied using genus‐level abundance data. In (G) and (H), the Spearman correlation of significantly enriched genus in the ethanol‐exposed groups was selected for correlation analysis. Results are expressed as mean ± SEM. *Asterisks indicate statistical significance (*p < 0.05, **p < 0.001, ***p < 0.001. ****p < 0.0001). In (D) and (E), * indicates SWITCH + H_2_O vs. SWITCH + EtOH; # indicates AING + EtOH vs. SWITCH + EtOH.

All colonic microbiota analyses were performed on colonic contents collected immediately after euthanasia at the end of T2. Distinct colonic microbiota profiles were observed between mice with high ethanol preference and consumption (SWITCH + EtOH group) and those with ethanol aversion (AING + EtOH group). Statistically significant increases in alpha diversity indexes were observed in the SWITCH + EtOH group compared with both AING + EtOH and SWITCH + H_2_O (AING + EtOH vs. SWITCH + EtOH: Shannon *p* = 0.0236, Pielou's *p* = 0.0048; SWITCH + H_2_O vs. SWITCH + EtOH: Shannon *p* = 0.0081, Pielou's *p* = 0.0009). However, no significant differences were detected between the AING + H_2_O and AING + EtOH groups (Figure 3A,B). Regarding beta diversity, significant dissimilarities were observed between ethanol‐exposed groups (*p* = 0.012) (Figure 3C).

At the phylum level, the SWITCH + EtOH group exhibited a significantly higher proportion of Bacillota (54.2%, *p* = 0.0133) and Actinomycetota (7.0%, *p* = 0.035) and a lower proportion of Bacteroidota (38.7%, *p* = 0.0014) compared with SWITCH + H_2_O (Figure 3D). At the family level, this group displayed a lower proportion of Muribaculaceae (38.7% vs. SWITCH + H_2_O, *p* = 0.0012), Erysipelatoclostridiaceae (1.1%, vs. SWITCH + H_2_O, *p* = 0.0317), Eubacterium (1.2%, vs. SWITCH + H_2_O, *p* = 0.005), and Ruminococcaceae (0.9%, vs. SWITCH + H_2_O, *p* = 0.0095) and a higher proportion of Lachnospiraceae (26.4% vs. AING + EtOH, *p* = 0.0139), Clostridiaceae (2.9%, vs. SWITCH + H_2_O, *p* = 0.0452), and Atopobiaceae (6.9%, vs. SWITCH + H_2_O, *p* = 0.0096) (Figure 3E). Linear discriminant analysis (LDA) of ethanol‐exposed mice identified taxa significantly associated with the microbiota of the SWITCH + EtOH group, including Lachnospirales, *Blautia*, Atopobiaceae, Coriobacteriaceae, Coriobacteriales, Coriobacteria, Actinobacteriota, *Colidextribacter, Tuzzerella, Enterorhabdus*, and *Lactococcus* (Figure 3F). In contrast, the taxa Erysipelatoclostridiaceae, *Marvinbryatia, Faecalibaculum*, Muribaculaceae, Bacteroidales, and Bacteroidia were more associated with the other groups (Figure 3F). Supporting this finding, *Marvinbryatia* exhibited a strong negative correlation with ethanol preference (*r* = −81.92, *p* = 0.0058), whereas *Blautia* showed a strong positive correlation (*r* = 84.24, *p* = 0.0037) (Figure 3G,H).

### The inferred functional profile of the colonic microbiota reveals reduced AA metabolism and biosynthesis, alongside increased secondary bile acid synthesis, in SWITCH + EtOH mice

**FIGURE 4:**
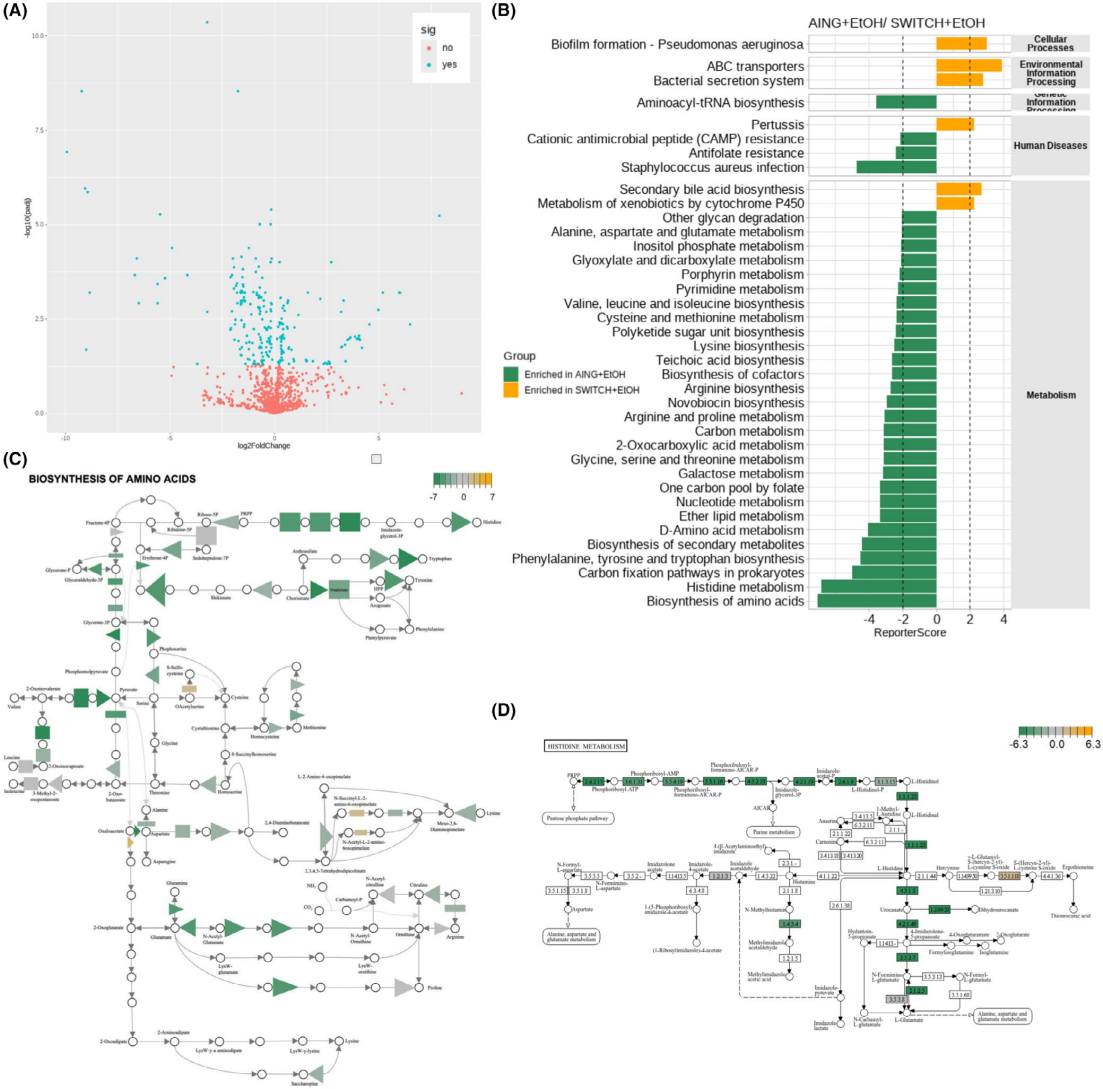
Microbiota functional metabolic inference at the T2 endpoint. (A) Volcano plot of all significantly different KO IDs. In the comparison between AING + EtOH and SWITCH + EtOH, (B) ReporterScore results, (C) biosynthesis of, and (D) histidine metabolism highlighting KOs with the highest and lowest *Z*‐scores.

Inferred functional microbiota metabolite analysis reveals significant shifts in microbiota metabolic pathways between ethanol‐averse (AING + EtOH) and ethanol‐preferring (SWITCH + EtOH) groups. Using DESeq2, we controlled for the interaction between diet and alcohol in the statistical analysis to isolate the effects of dietary changes and alcohol consumption. This approach identified 889 differentially expressed bacterial genes out of a total of 5987, specifically those with a fold change greater than 1 or less than −1, as exemplified in Figure 4A.

Further analysis using ReporterScore, integrating DESeq2 *p*‐values, allowed the identification of enriched metabolic pathways in the colonic microbiota of ethanol‐exposed groups (Figure 4B). There was a significant reduction in AA biosynthesis (*ReporterScore* = −7.546344500, *Z*‐score = −25.92, *p*.adj = 0.0267), which had the lowest ReporterScore values along with many other pathways related to biosynthesis of AA (Figure 4C), such as histidine metabolism (Figure 4D), phenylalanine, tyrosine, and tryptophan metabolism, and D‐amino acid production. In contrast, the bacterial metabolic pathways with the highest enrichment scores (*p* < 0.05) were ABC transporters, the bacterial secretion system, and secondary bile acid synthesis (Figures S1–S3).

### Network analysis reveals the interaction between metabolic pathways and the most representative genes in the Switch + EtOH mice

**FIGURE 5:**
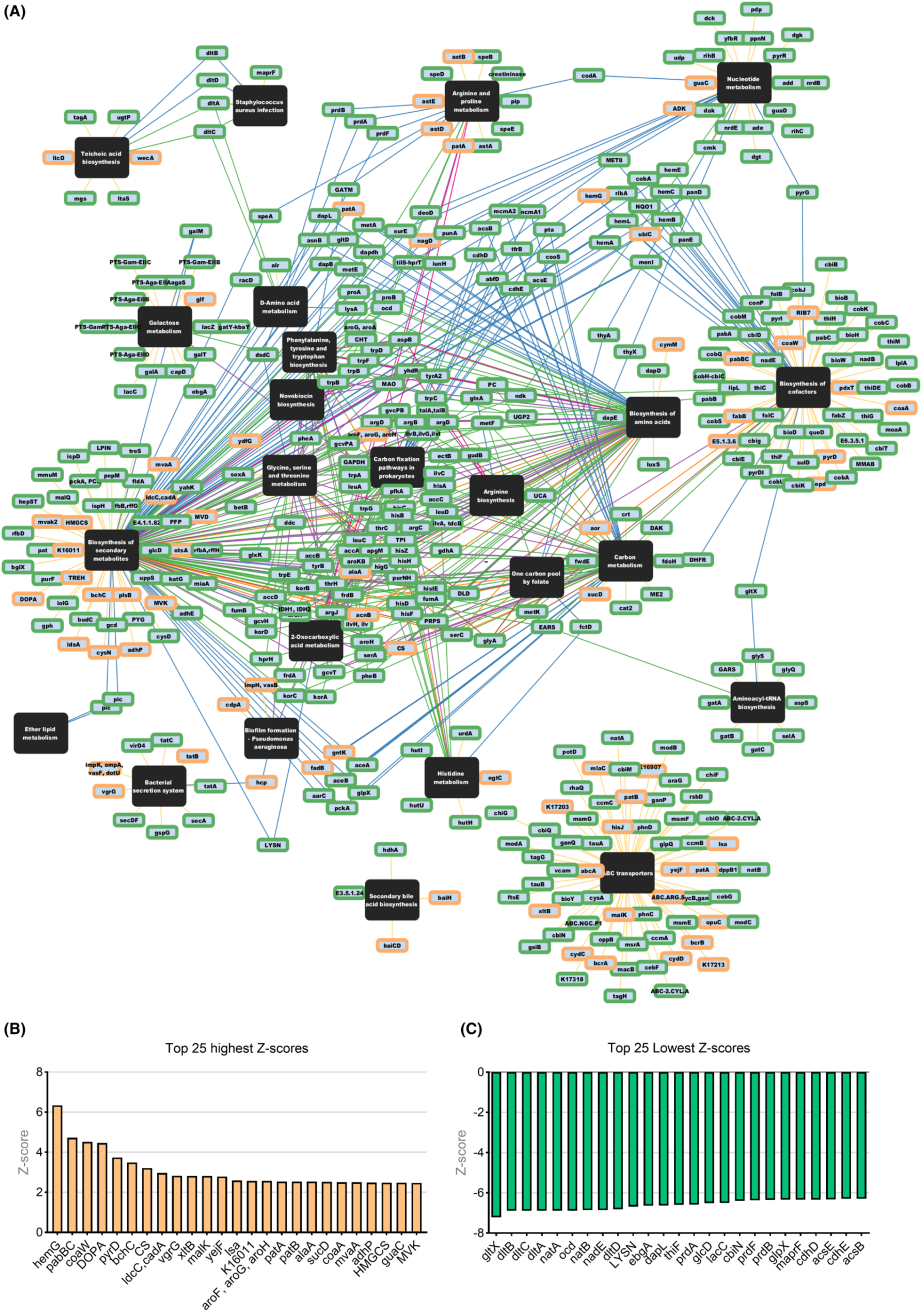
Network‐based metabolic inference differentiating ethanol‐averting and ethanol‐preferring mice at the T2 endpoint. (A) Network of significant KOs in the AING + EtOH vs. SWITCH + EtOH comparison, displaying the Top 25, (B) lowest and (C) highest *Z*‐scores. The network (A) was constructed using the ReporterScore R package, incorporating all significantly enriched metabolic pathways and KO IDs. Green nodes represent depleted KOs, while yellow nodes represent enriched KOs in this comparison. Edge colors indicate node degrees: yellow= 1, blue = 2, green = 3, purple = 4, orange = 5, brown = 6, pink = 7. Network‐associated scores were computed using the MetaNet R package. *Z*‐scores (B) and (C) were calculated using standard parameters in the ReporterScore package.

Although rapidly absorbed, chronic ethanol intake allows a portion of alcohol and its metabolites to reach the colon, directly impacting microbial communities, while also exerting systemic effects that disrupt host homeostasis. In this context, network‐based analyses identified microbiota genes and pathways associated with persistent alcohol consumption behavior and ethanol‐induced physiological imbalance. Many KO IDs identified in the inferred metabolome participate in multiple enriched pathways. The network (Figure 5A) integrates significantly enriched pathways linked to KO IDs differentially abundant in AING + EtOH vs. SWITCH + EtOH groups, highlighting interconnected processes. Genes with highest betweenness and closeness scores were mainly involved in essential AA biosynthesis (*lysA, trpE, trpG, glyA, serC*, and *metK*) and energy metabolism (*ndk, DLD*, and *UGP2*). Oxidative stress‐related genes (*hcp, EARS*, and dltA) were also prominent (Tables 1 and 2). Central genes (high degree centrality) included aminotransferases for aspartate, glycine, serine metabolism (*aspB, glyA*, and *serC*), TCA cycle enzymes (*IDH1, IDH2*, and *acnB*), and histidine biosynthesis (*hisC*) (Table 3).

**TABLE 1:** Top 10 genes with highest betweenness score.

Genes	Function	Betweenness	*p* adj	*Z*‐score
*lysA*	Lysine biosynthesis	4967	0.02093595	−2.034791
*hcp*	Complex protein transport mechanism	3620	0.020408	2.045394
*EARS*	Aminoacylate tRNA with glutamate	3360	1.067629E‐6	−4.740182
*UGP2*	Glucose‐1‐phosphate + UTP into UDP‐glucose	2878	7.286304E‐4	−3.183063
*pfkA*	Fructose‐6‐phosphate to fructose‐1,6‐bisphosphate	2538	8.007553E‐4	−3.155631
*trpE*	Biosynthesis of the amino acid tryptophan	2527.28	8.907992E‐4	−3.124414
*trpG*	Biosynthesis of the amino acid tryptophan	2527.28	0.001293635	−3.012944
*glyA*	Interconversion of glycine and serine	2236	0.007503671	−2.432202
*ndk*	Transfer of phosphates from NTPs to NDPs	2006	2.666088E‐4	−3.463489
*dltA*	Membrane protein involved in the export of D‐alanine	1983.5	2.821249E‐12	−6.889783

**TABLE 2:** Top seven genes with highest closeness score.

Genes	Function	Closeness	*p* adj	*Z*‐score
*glyA*	Interconversion of glycine and serine	0.00102459	0.007503671	−2.432202
*serC*	3‐phosphohydroxypyruvate to 3‐phosphoserine	0.00101626	1.998585E‐7	−5.069092
*DLD*	Component of the pyruvate dehydrogenase complex	0.001004016	0.006438649	−2.487145
*ndk*	Phosphate transferase from NTPs to NDPs	9.92E+02	2.666088E‐4	−3.463489
*UGP2*	Glucose‐1‐phosphate + UTP into UDP‐glucose	9.80E+02	7.286304E‐4	−3.183063
*EARS*	Aminoacylate tRNA with glutamate	9.65E+01	1.067629E‐6	−4.740182
*metK*	Met+ATP into S‐adenosylmethionine (SAM)	9.63E+02	2.993366E‐5	−4.013333

**TABLE 3:** Genes that have five or more degrees in the network.

Genes	Function	Degrees	*p* adj	*Z*‐score
*yhdR*	Aspartate aminotransferase	7	0.005067343	−2.5712
*aspB*	Aspartate aminotransferase	7	2.310839E‐4	−3.501776
*glyA*	Interconversion of glycine and serine	6	0.007503671	−2.432202
*serC*	Phosphoserine aminotransferase, key pathway of serine biosynthesis	5	1.998585E‐7	−5.069092
*IDH1, IDH2*	Isocitrate dehydrogenase	5	0.002090999	−2.864097
*acnB*	Oxidation of propionate to pyruvate in *Escherichia coli*	5	0.01011598	2.322018
*hisC*	Biosynthesis of histidine	5	1.488824E‐7	−5.124856

The top 25 highest *Z*‐scores (Figure 5B) indicate an increased representation of pathways related to bacterial metabolism, oxidative‐stress response, and dopamine metabolism in the microbiota of the SWITCH + EtOH group. Genes more prominently represented in the network included *hemG* (heme biosynthesis), *bchC* (tetrapyrrole metabolism), and *ahpF* (oxidative‐stress defense). Sugar transport and metabolism‐related genes were also more represented, including *sc‐EIIC* (sugar transport), *uhpC* (phosphate transport), and *ascF* (ascorbate metabolism). Additionally, genes potentially linked to dopamine metabolism and neurotransmitter interactions were more represented in the network, including *DOPA* (dopamine biosynthesis and degradation), *padBC* (a dopamine precursor), and *ttdA* (aromatic acid metabolism associated with neurotransmission).

Conversely, the top 25 lowest *Z*‐scores (Figure 5C) indicate a significant suppression of AA biosynthesis and transport, with lower representation in the SWITCH + EtOH group microbiota of genes, such as *dapL* (lysine biosynthesis), *lysN, lysP* (lysine transport), *sstT* (serine and threonine transport), and *prdA, prdD, prdE* (proline metabolism and propionate production). Additionally, *mapA* (AA degradation), *natA*, and *natB* (protein acetylation) were less represented in the network. Genes associated with SCFA production also showed reduced representation, including *prdA, prdD, prdE* (proline conversion to propionate), *ocd* (fatty acid metabolism and butyrate production), and *GLPF* (glycerol transport for microbial fermentation).

### The quantification of SCFAs and AA validates the functional inferences

**FIGURE 6:**
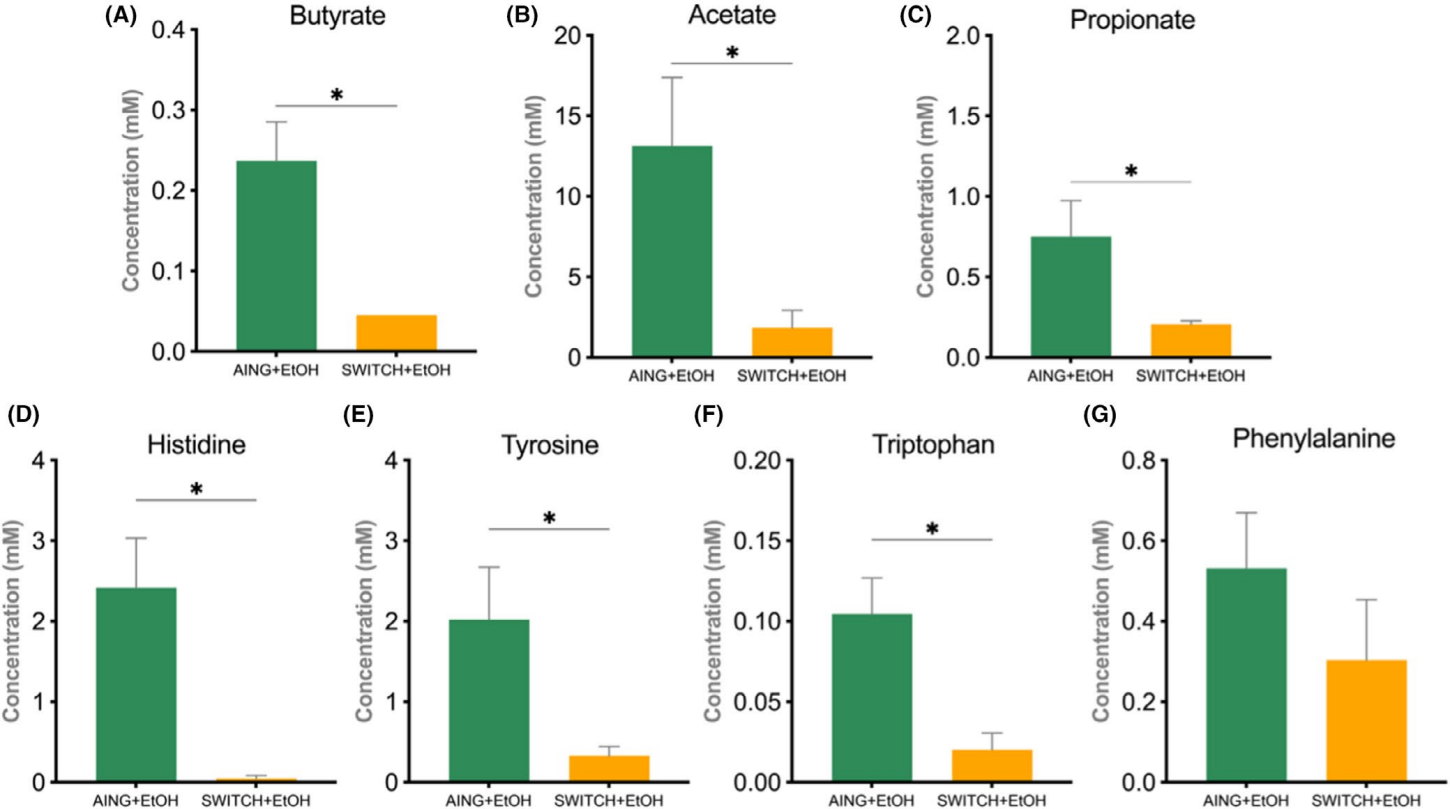
Short‐chain fatty acid and amino acids concentration comparison between AING + EtOH and SWITCH + EtOH at the T2 endpoint. Concentration of (A) butyrate, (B) acetate, (C) propionate, (D) histidine, (E) tyrosine, (F) tryptophan, and (G) phenylalanine. Mann–Whitney statistical test was performed for all comparisons. Results are expressed as mean ± SEM. *Asterisks indicate statistical significance.

The quantification of SCFAs validates the functional inferences, given that colonic content showed decreased levels of butyrate (*p* = 0.0476), acetate (*p* = 0.0317), and propionate (*p* = 0.0159) in the SWITCH + EtOH group compared with the AING + EtOH group (Figure 6A–C). A similar pattern was observed for the AA histidine (*p* = 0.0159), tyrosine (*p* = 0.0159), and tryptophan (*p* = 0.0317), whereas phenylalanine did not exhibit significant differences (Figure 6D–G).

## DISCUSSION

Previously, our research group demonstrated that switching mice from a highly palatable HSB diet, which is already associated with binge‐eating behavior, to a standard AING diet and subsequently exposing them to a free‐choice alcohol paradigm induces high ethanol consumption and preference (Júnior et al., 2025; Martins de Carvalho et al., 2018; Moreira Júnior et al., 2019, 2021, 2023). In contrast, animals continuously maintained on the AING diet exhibited aversion to alcohol (Martins de Carvalho et al., 2018; Moreira Júnior et al., 2019). Within this context, we have reported alterations in transcriptional gene regulation of GABA and dopamine receptors in the PFC and striatum, neuroinflammation, systemic inflammation, behavioral changes, and modifications in colonic microbiota structure, which appear to both result from and influence the drive toward high ethanol consumption and preference (Martins de Carvalho et al., 2018; Moreira Júnior et al., 2019, 2023). It is noteworthy that, in previous studies employing a free‐choice ethanol consumption paradigm, animals maintained on the HSB diet throughout the entire experimental period did not show increased ethanol intake or preference, and also did not display aversion (Martins de Carvalho et al., 2018; Moreira Júnior et al., 2019, 2023). In the present study, using animals that replicate this previously established model, we provide additional evidence supporting the involvement of colonic microbiota in alcohol‐related behaviors by analyzing compositional and functional features based on inferred intestinal microbiota metabolomics. Specifically, we compared animals exhibiting alcohol aversion (AING + EtOH) with those showing high alcohol preference (SWITCH + EtOH). Importantly, all colonic microbiota analyses were obtained only at the T2 endpoint; the observed alterations could also reflect adaptations to 4 weeks of ethanol exposure superimposed on the metabolic imprint of the prior HSB diet.

The colonic microbiota has been implicated in modulating both ethanol preference and aversion. Ezquer et al. (2021) showed that antibiotic administration in rats with high alcohol consumption reduced voluntary ethanol intake by 70%, suggesting a direct role of the gut microbes in regulating this behavior (Ezquer et al., 2021). Similarly, Carbia et al. (2023) identified a specific microbial signature in individuals with excessive alcohol consumption, characterized by associations with craving and compulsive drinking, in contrast to profiles observed in abstinent or moderate drinkers (Carbia et al., 2023). The present study observed distinct microbial profiles in animals exhibiting high ethanol preference compared with those showing aversion. *Marvinbryantia* correlated negatively with ethanol preference, whereas *Blautia* showed a positive correlation. *Marvinbryantia* is a SCFA producer, particularly butyrate, which exerts neuroprotective effects, attenuates microglia‐mediated neuroinflammation, and modulates gut microbial communities (Simon‐O'Brien et al., 2015). These properties may contribute to improved cognitive and behavioral outcomes related to alcohol exposure and reduced risk of relapse. Thus, the diminished abundance of *Marvinbryantia* may indicate increased vulnerability to compulsive alcohol use. Conversely, *Blautia* has been associated with metabolic conditions, such as obesity and chronic inflammation, factors potentially enhancing susceptibility to alcohol abuse (Liu et al., 2017; Wang et al., 2021). Consistently, González‐Zancada et al. (2020) reported elevated *Blautia* levels in regular beer consumers, and Wang et al. (2021) found a positive association between Blautia abundance and alcohol exposure in female mice (González‐Reimers et al., 2014; Wang et al., 2021). Interestingly, Samulėnaitė et al. (2024) reported a reduced relative abundance of *Blautia wexlerae* in individuals with food addiction (Samulėnaitė et al., 2024). In mice, supplementation with *B. wexlerae* or prebiotics promoting its growth, such as lactulose and rhamnose, led to significant improvements in food addiction‐like behaviors (Samulėnaitė et al., 2024). These observations suggest that *Blautia* may play a complex, context‐dependent role in addictive behaviors, potentially exerting distinct effects on food and substance‐related addictions. Collectively, these findings indicate that colonic microbiota alterations likely influence the physiological mechanisms regulating alcohol consumption, contributing to either vulnerability or resilience to compulsive drinking.

Considering the functional interplay between microbiota and host metabolism, shifts in microbiota composition may profoundly impact systemic metabolic processes (Fan & Pedersen, 2021). Microbiota‐derived metabolites, particularly those involved in AA metabolism, influence neurotransmitter availability and neuronal functions implicated in addiction (Chen & Xu, 2021; Lin et al., 2017). In our model, animals from the SWITCH + EtOH group exhibited significant reductions in AA metabolism. Yang et al. (2021) reported decreased serum levels of BCAAs, cysteine, and methionine in ethanol‐exposed rats, which were associated with increased tryptophan availability, altered serotonin synthesis, reduced antioxidant capacity, and dysbiosis, highlighting the microbiota's role in AA metabolism and alcohol dependence (Yang et al., 2021). In humans, Yang et al. (2024) found reduced BCAA, glutamate, and aspartate levels in the serum and urine of excessive drinkers, reflecting disrupted AA metabolism, oxidative stress, and increased risk of liver dysfunction (Yang et al., 2024). Similarly, Walter et al. (2008) observed lower plasma levels of GABA and serine in alcohol‐dependent individuals (Walter et al., 2008). The gut microbiota contributes to AA metabolism by producing essential AAs, such as lysine and tryptophan, and synthesizing nonessential AAs, including alanine, proline, and glutamate (Agus et al., 2018; Li et al., 2024). These microbial activities influence systemic metabolism and neurotransmission by modulating the availability of AA precursors to the liver and brain (Agus et al., 2018; Li et al., 2024). For instance, bacteria such as *Bifidobacterium* can convert glutamate into the inhibitory neurotransmitter GABA (Duranti et al., 2020). Interestingly, studies using the same model reported reduced GABA receptor expression in the PFC and decreased *Bifidobacterium* abundance in animals with high alcohol preference (Martins de Carvalho et al., 2018; Moreira Júnior et al., 2019, 2021). Together, these findings underscore the role of gut microbiota in modulating AA metabolism and its potential contribution to alcohol use disorder pathophysiology.

Another AA whose colonic metabolism is reduced in the high alcohol preference group in our study is histidine, a histamine precursor. Vanhanen et al. (2013) showed that deficient brain histamine impairs H_3_ receptor‐mediated inhibition of alcohol reward, underscoring its modulatory role in alcohol‐related behaviors (Vanhanen et al., 2013). In addition, gut microbiota can influence systemic histamine levels via microbial histidine decarboxylation, linking dysbiosis to altered histamine availability and behavioral outcomes related to alcohol use (De Palma et al., 2022; Rastelli et al., 2019). These findings support the hypothesis that reduced brain histamine contributes to the elevated ethanol preference observed in the SWITCH + EtOH group, likely due to impaired inhibitory signaling in reward circuits (Vanhanen et al., 2013). H_3_ receptors also form heterodimers with dopamine D_1_ and D_2_ receptors, modulating dopaminergic transmission and influencing reward processing (Ellenbroek, 2013). As a key mediator of addiction, dopamine reinforces alcohol intake and facilitates persistence and relapse of alcohol‐seeking behavior (Söderpalm & Ericson, 2024). Using the same animal model of the present study, Martins de Carvalho et al. (2018) reported *Drd2* upregulation in the nucleus accumbens (NAc) of the SWITCH + EtOH group compared with AING + EtOH, while in the PFC, *Drd1* was upregulated and *Drd2* downregulated (Martins de Carvalho et al., 2018). This suggests a mesocorticolimbic dopamine imbalance, characterized by a hyperdopaminergic NAc promoting alcohol‐seeking and a hypodopaminergic PFC linked to cognitive deficits and impaired inhibitory control (Koob & Volkow, 2016). Consistently, we found enrichment of microbial genes involved in L‐DOPA synthesis and aromatic amino acid metabolism (*dopa decarboxylase*, *padBC*, and *ttdA*), suggesting that gut dysbiosis may modulate central dopamine tone via gut–brain axis pathways (Hamamah et al., 2022; Qu et al., 2024). Systematic reviews by Qu et al. (2024) and Mhanna et al. (2024) reinforce the potential role of the microbiota in modulating dopaminergic pathways and drug‐seeking behavior (Qu et al., 2024). Supporting this, Ezquer et al. (2022) showed that *Lactobacillus rhamnosus GG* alters dopamine transporter expression and dopamine clearance in alcohol‐preferring rats (Ezquer et al., 2022). Together, these findings suggest that gut microbiota alterations may contribute to the dopaminergic dysregulation observed in this model of alcohol use disorder.

Our analysis revealed increased microbial genes associated with tryptophan metabolism and ABC transporter pathways in alcohol‐exposed animals. ABC transporters play a central role in the uptake and export of tryptophan and its derivatives, suggesting altered microbial handling of this essential amino acid under chronic alcohol exposure (Bilsing et al., 2023; Ranhotra, 2024; Vidal et al., 2020). This shift may reflect a broader microbial metabolic adaptation affecting host–microbiota interactions, particularly in pathways dependent on tryptophan availability and its downstream metabolites (Lobionda et al., 2019; Ranhotra, 2024; Wrzosek et al., 2021). An increased microbial metabolic pathway in mice with high alcohol preference is the synthesis of secondary bile acids, which occurs in the intestine through the bacterial metabolism of primary bile acids (Heinken et al., 2019). Taxa associated with the SWITCH + EtOH group in the LEfSe analysis, such as *Lachnospirales*, include bacteria capable of performing 7α‐dehydroxylation, the main pathway for converting primary into secondary bile acids, leading to the formation of deoxycholic acid (DCA) and lithocholic acid (LCA) (Ramírez‐Pérez et al., 2017; Ridlon et al., 2015; Zeng et al., 2020). Additionally, *Blautia* contributes to bile acid metabolism, while Coriobacteriaceae, including *Eggerthella* and *Collinsella*, are known for modifying bile acids through oxidation and epimerization reactions (Astbury et al., 2020; Heinken et al., 2019; Ridlon & Gaskins, 2024; Wegner et al., 2017). Studies indicate that alcohol can affect the regulation of bile acid receptors, such as FXR and TGR5, reducing the negative feedback of bile secretion and increasing the primary bile acids available for bacterial modification (Chiang & Ferrell, 2020; Spatz et al., 2021), likely explaining the observed increase in secondary bile acid synthesis pathway in our study. The production of DCA and LCA is known to have pro‐inflammatory effects, potentially increasing intestinal permeability, findings that have been observed in this model in previous studies (Moreira Júnior et al., 2019, 2023; Ridlon et al., 2015; Spatz et al., 2021).

Finally, SCFAs appear to have significant metabolic relevance in high ethanol consumption and preference. Firstly, the reduction in AA metabolism and biosynthesis compromises the availability of these compounds for SCFA synthesis (Louis & Flint, 2017). Additionally, the low expression of genes such as *prdA*, *prdD*, and *prdE*, involved in the conversion of proline into propionate, *ocd*, essential for fatty acid metabolism and butyrate synthesis, and *glpF*, essential for glycerol transport and its utilization in bacterial fermentation for SCFA production, may contribute to this reduction (Facchin et al., 2024; Louis & Flint, 2017; Nie et al., 2019; Ramos Meyers et al., 2022). These metabolites, such as butyrate and propionate, play protective roles in maintaining intestinal‐barrier integrity, as well as exerting hepatoprotective, immunomodulatory, and neuroprotective effects by regulating gene expression in brain regions such as the striatum and PFC cortex (den Besten et al., 2013; Facchin et al., 2024; Hamamah et al., 2022). Since AUD is often associated with increased intestinal permeability, liver dysfunction, immunosuppression, neuroinflammation, and behavioral alterations, the reduction in SCFA production may directly influence these pathological processes (Shen et al., 2024). In this context, Tierney et al. (2023) evaluated the impact of alcohol consumption and antibiotic use on SCFA production in a human intestinal model simulating the luminal and mucosal colonic environment (Tierney et al., 2023). Their findings showed that both treatments reduced SCFA production, including acetate, propionate, and butyrate. However, supplementation with a microbial symbiotic restored and enhanced intestinal function, increasing butyrate and acetate levels, suggesting that dietary or probiotic interventions may help restore gut microbiome function following alcohol‐induced disturbances (Tierney et al., 2023). Quintanilla et al. (2024) investigated the effects of intragastric administration of acetate, propionate, and butyrate in alcohol‐preferring rats (Quintanilla et al., 2024). The results indicated that this intervention significantly reduced voluntary ethanol consumption and was associated with lower intestinal and hepatic inflammation and attenuation of glial activation in the central nervous system (Quintanilla et al., 2024). In mice, Bokoliya et al. (2024) orally administered the SCFA valeric acid, which has a structure like GABA, for 10 days. The authors observed a 40% reduction in excessive alcohol consumption, a 53% decrease in blood ethanol concentration, and an improvement in anxiety‐related behavior without affecting overall food and water intake (Bokoliya et al., 2024). These results emphasize the significant role of SCFAs in regulating alcohol preference, highlighting their metabolic influence on neurobiological pathways involved in ethanol consumption.

This study's findings reinforce the complex interaction between colonic microbiota, metabolism, and the neurobiological mechanisms that may contribute to compulsive alcohol consumption. Mice that escalated their ethanol intake after HSB diet withdrawal exhibited a distinct colonic microbiota, reduced SCFA output, altered AA‐derived metabolites, and enrichment of KEGG orthologs linked to oxidative‐stress defense, bile acid conversion, and dopamine‐related pathways. These findings corroborate our previous observations in the same model (Martins de Carvalho et al., 2018; Moreira Júnior et al., 2019, 2021, 2023) and suggest that microbiota‐derived metabolites may influence neuroinflammation and dopaminergic balance. Although many functional insights were obtained through in silico inference, they were supported by direct quantification of key metabolites, strengthening confidence in the predicted pathways. However, some limitations warrant consideration. Because all microbiota and metabolite measurements were taken once, on colonic contents collected at the T2 endpoint, the study offers only a single cross‐section and cannot ascertain whether microbial shifts precede or follow the escalation in drinking. Future studies should pair longitudinal metagenomic and targeted metabolomic sampling across baseline, the eight‐week HSB phase (T1), withdrawal, and the 4‐week drinking period (T2) with selective modulation of candidate taxa or metabolites. Fecal microbiota transplantation from HSB‐withdrawn, ethanol‐preferring donors into naïve recipients would also provide a stringent test of causality. Despite these inherent challenges, the integration of behavioral, metabolic, and microbial data in the present study offers valuable mechanistic insight and highlights microbiota‐derived metabolites as promising therapeutic targets for AUD.

## AUTHOR CONTRIBUTIONS

MVM conducted bioinformatics and statistical analyses and processed the short‐chain fatty acid and amino acid quantification material. Additionally, MVM prepared all graphical representations, interpreted and organized the results, and wrote the initial manuscript. TCL and MSA contributed to analyzing colonic microbiota composition, result interpretation, and discussion writing. JSO and FLAB contributed to the quantification of short‐chain fatty acids and amino acids by HPLC. REMJ conducted the animal model, performed total bacterial DNA extraction and 16S rRNA gene sequencing, processed the material for short‐chain fatty acid and amino acid quantification, interpreted the results, wrote the discussion, and supervised the writing of the entire initial manuscript. GB contributed to the interpretation of the results, discussion, and revision of the initial manuscript. ALBG coordinated the experiments, analyzed the results, and supervised the manuscript development phase. All authors participated in the manuscript revision and approved the final version.

## FUNDING INFORMATION

This study was supported by the Fundação de Amparo à Pesquisa do Estado de Minas Gerais (FAPEMIG, grant number: APQ‐045517‐22 and APQ‐03984‐24), Instituto Nacional de Ciências e Tecnologia sobre Substâncias Psicoativas (INCT‐SP)/Conselho Nacional de Desenvolvimento Científico e Tecnológico (CNPq) (grant number 406958/2022), the Coordenação de Aperfeiçoamento de Pessoal de Nível Superior (CAPES), the Pró‐Reitoria de Pesquisa—UFMG (PRPq‐UFMG), and the Pós‐Graduação em Genética—ICB—UFMG, Brazil.

## CONFLICT OF INTEREST STATEMENT

The authors declare that this research was conducted without any commercial or financial relationships that could be construed as a potential conflict of interest.

## Supporting information


Figures S1–S3

## Data Availability

The data that support the findings of this study are available from the corresponding author upon reasonable request.
