# Supplementary material for: Predicted functional alterations in colonic microbiota metabolism underlie ethanol consumption and preference behavior in mice
Source: Alcohol Clin Exp Res (Hoboken). 2025 Oct 30;49(11):2436–50. doi: 10.1111/acer.70165 (PMC12638287; doi:10.1111/acer.70165)
Supplement: Supplementary file 1 — Figures S1–S3 [file ACER-49-2436-s001.docx]

**SUPPLEMENTARY METHODOLOGY**

**Total Bacterial DNA Extraction and 16S rRNA Gene Sequencing**

Total bacterial DNA was extracted from colon contents using the QIAamp® DNA Stool Mini Kit (QIAGEN®, Germany), following the manufacturer’s instructions. The extracted DNA was amplified using primers specific to the V3–V4 region of the 16S rRNA gene. Amplicons were then purified with AMPure XP beads (Beckman Coulter, USA), and their concentrations were estimated using the PicoGreen dsDNA assay (Invitrogen, USA). Subsequently, the amplicons were normalized based on DNA concentration, pooled into libraries, and accurately quantified by qPCR using the KAPA Library Quantification Kit for Illumina platforms (KAPA Biosystems, USA). Next-generation sequencing was performed on an Illumina MiSeq platform.

**16S Microbiome Profiling and OTU Clustering Pipeline**

Raw FASTQ datasets were pre-processed using Trimmomatic v0.39 (Bolger et al., 2014). Illumina adapters were removed, and leading and trailing bases with a quality score below three were trimmed. A 4-base sliding window was then used to trim reads with an average quality below 20, and any reads shorter than 100 bp were discarded (Douglas et al., 2020). Subsequent filtering, analysis, and data exploration were performed using the Nephele platform from the National Institute of Allergy and Infectious Diseases (NIAID) Office of Cyber Infrastructure and Computational Biology (OCICB) in Bethesda, MD (Weber et al., 2018).

**Functional Profiling Inference and Differential Pathway Analysis in Microbiome Data**

Abundance frequency tables from processed reads were analyzed in QIIME2 v2023.7 to calculate alpha (Shannon and Pielou) and beta (unweighted Unirac distance) diversity indices (Hall and Beiko, 2018). Alpha diversity was compared using a two-way ANOVA (GraphPad prism v10.3.1), while beta diversity was assessed via Adonis (R package Vegan v 2.6-10) (Kers and Saccenti, 2021, Oksanen et al., 2017). Linear Discriminant Analysis Effect Size (LEfSe) v 1.1.2 identified differentially abundant taxa among groups (Chang et al., 2022). For functional inference, abundance frequency coupled with taxonomic identification tables were mapped to KEGG orthologs (KOs) using PICRUSt2 v2.5.2, and differential expression analysis was performed in DESeq2 between AING+EtOH and SWITCH+EtOH groups, focusing on differences between ethanol aversion and high ethanol preference (Douglas et al., 2020, Kanehisa et al., 2016, Love et al., 2014, Srivastava et al., 2024). The resulting p-values were integrated into the standard pipeline available in the R package ReporterScore v 0.1.9 to detect enriched or suppressed metabolic pathways, which were also individually visualized in the R package Pathview v 3.20 (Luo et al., 2017, Peng et al., 2024). Lastly, MetaNet v 0.1.0 R package was used to calculate centrality parameters and build the network nodes and edges based on the interactions among KOs and pathways. For centrality calculations both ABC transporters and Secondary bile acid synthesis were excluded due to their nature since none of them had associated metabolites that made connections to more than one pathway and could lead to non-representative results. The network was built and visualized in the program Cytoscape v 3.10.0 (Narang et al., 2014, Shannon et al., 2003). A significance level of p < 0.05 was adopted for all analyses.

**References**

1. Bolger AM, Lohse M, Usadel B (2014) Trimmomatic: a flexible trimmer for Illumina sequence data. Bioinformatics 30**:**2114-2120.

2. Chang F, He S, Dang C (2022) Assisted Selection of Biomarkers by Linear Discriminant Analysis Effect Size (LEfSe) in Microbiome Data. J Vis Exp.

3. Douglas GM, Maffei VJ, Zaneveld JR, Yurgel SN, Brown JR, Taylor CM, Huttenhower C, Langille MGI (2020) PICRUSt2 for prediction of metagenome functions. Nat Biotechnol 38**:**685-688.

4. Hall M, Beiko RG (2018) 16S rRNA Gene Analysis with QIIME2. Methods Mol Biol 1849**:**113-129.

5. Kanehisa M, Sato Y, Kawashima M, Furumichi M, Tanabe M (2016) KEGG as a reference resource for gene and protein annotation. Nucleic Acids Res 44**:**D457-462.

6. Kers JG, Saccenti E (2021) The Power of Microbiome Studies: Some Considerations on Which Alpha and Beta Metrics to Use and How to Report Results. Front Microbiol 12**:**796025.

7. Love MI, Huber W, Anders S (2014) Moderated estimation of fold change and dispersion for RNA-seq data with DESeq2. Genome Biol 15**:**550.

8. Luo W, Pant G, Bhavnasi YK, Blanchard SG, Brouwer C (2017) Pathview Web: user friendly pathway visualization and data integration. Nucleic Acids Res 45**:**W501-W508.

9. Narang P, Khan S, Hemrom AJ, Lynn AM, Consortium OSDD (2014) MetaNET--a web-accessible interactive platform for biological metabolic network analysis. BMC Syst Biol 8**:**130.

10. Oksanen J, Blanchet FG, Kindt R, Legendre P, Minchin PR, O’hara RB, Simpson G, Solymos P, Henry M, Stevens H (2017) Ordination methods, diversity analysis and other functions for community and vegetation ecologists. Vegan: Community Ecol Package**:**05-26.

11. Peng C, Chen Q, Tan S, Shen X, Jiang C (2024) Generalized reporter score-based enrichment analysis for omics data. Brief Bioinform 25.

12. Shannon P, Markiel A, Ozier O, Baliga NS, Wang JT, Ramage D, Amin N, Schwikowski B, Ideker T (2003) Cytoscape: a software environment for integrated models of biomolecular interaction networks. Genome Res 13**:**2498-2504.

13. Srivastava A, Akhter Y, Verma D (2024) A step-by-step procedure for analysing the 16S rRNA-based microbiome diversity using QIIME 2 and comprehensive PICRUSt2 illustration for functional prediction. Arch Microbiol 206**:**467.

14. Weber N, Liou D, Dommer J, MacMenamin P, Quiñones M, Misner I, Oler AJ, Wan J, Kim L, Coakley McCarthy M, Ezeji S, Noble K, Hurt DE (2018) Nephele: a cloud platform for simplified, standardized and reproducible microbiome data analysis. Bioinformatics 34**:**1411-1413.

**SUPPLEMENTARY FIGURES**


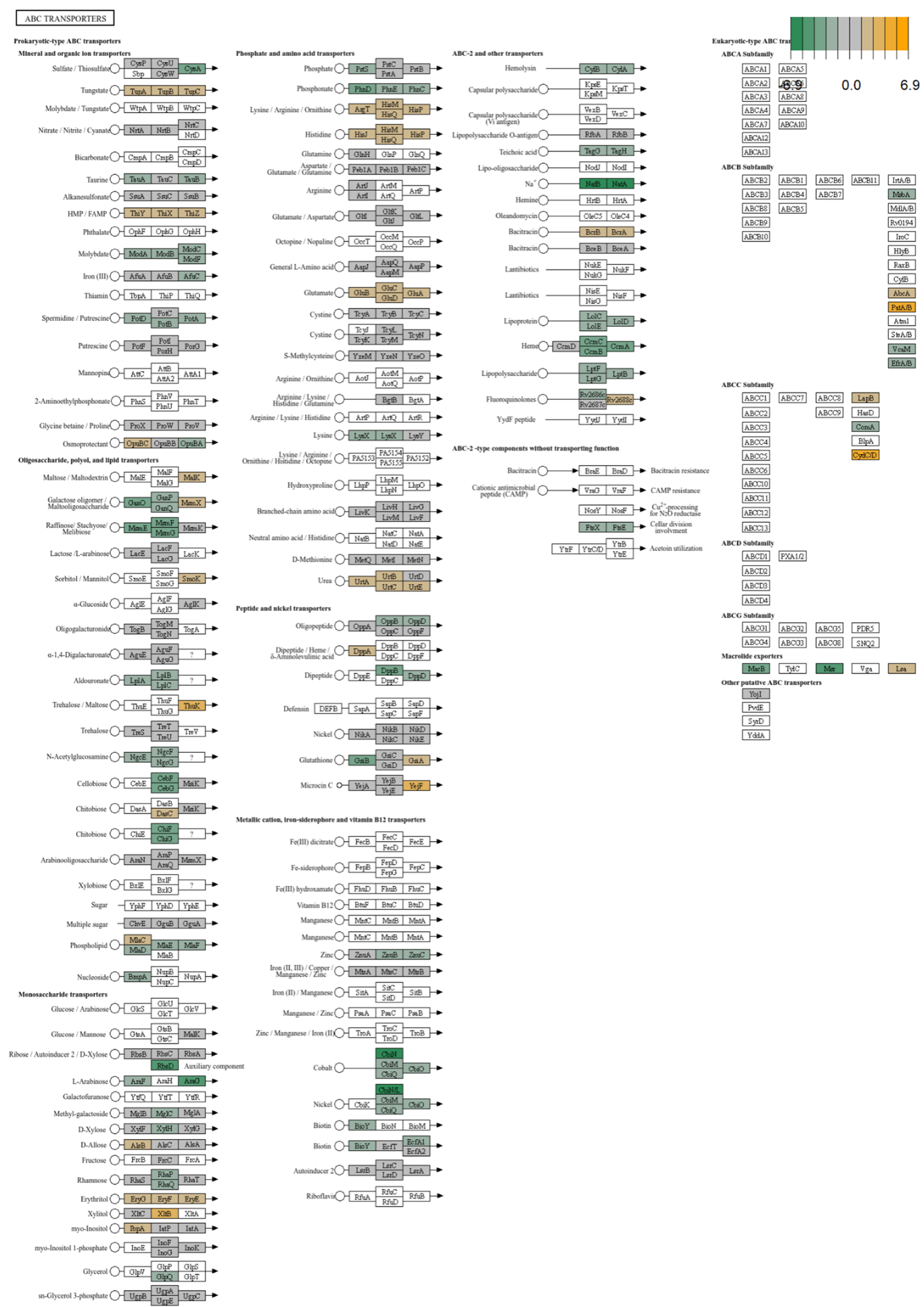


**Supplementary Figure 1**: ABC transporters


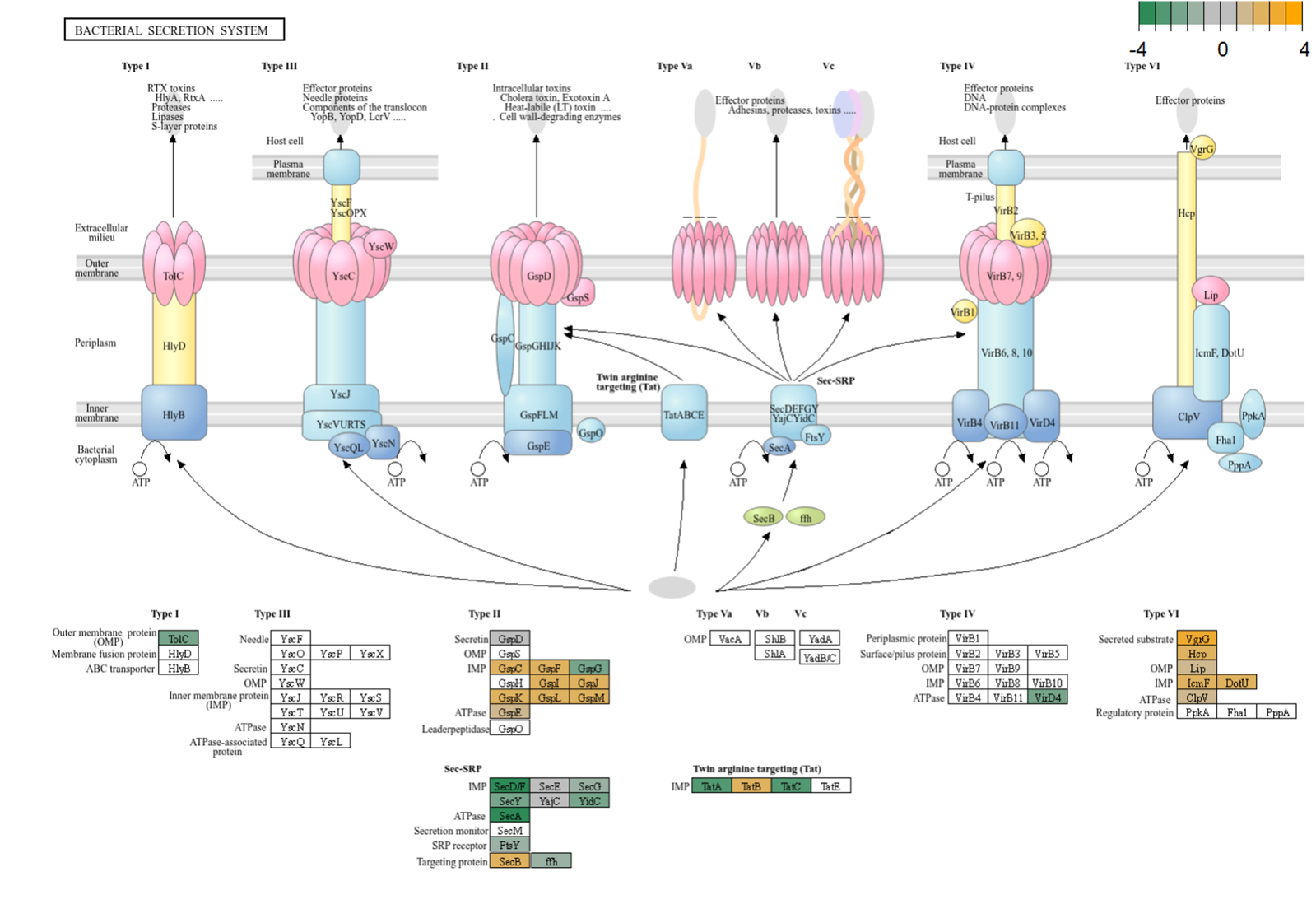


**Supplementary Figure 2**: Bacterial secretion system


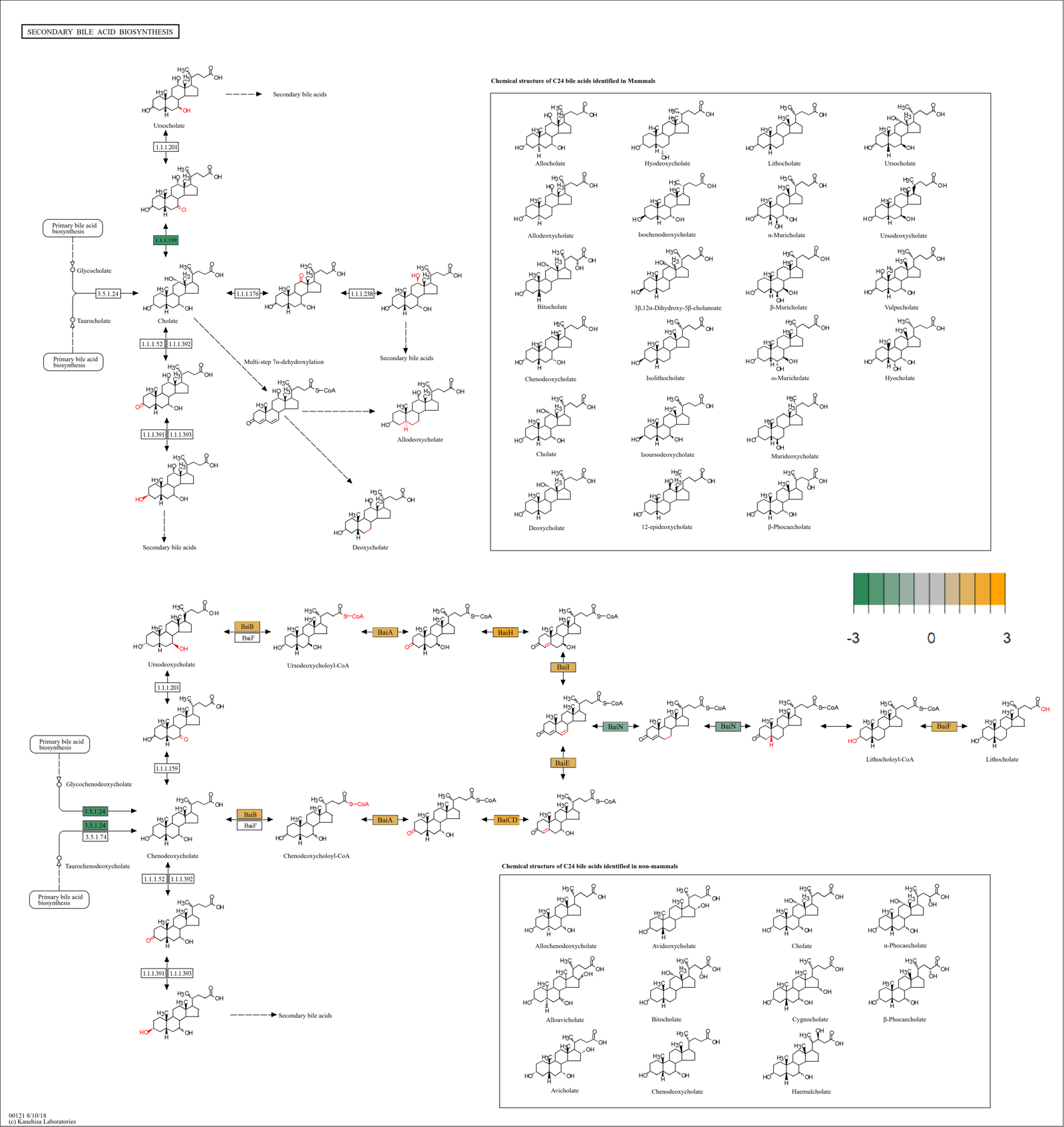


**Supplementary Figure 3:** **Secondary bile acid synthesis**
